# Screening the Resistance of Male *Aedes aegypti* and *Anopheles coluzzii* to Insecticides in the Context of Using Genetic Control Tools in Burkina Faso

**DOI:** 10.3390/insects16030315

**Published:** 2025-03-18

**Authors:** Hamidou Maiga, Abel Souro Millogo, Koama Bayili, Etienne Bilgo, Inoussa Toe, Roch Kounbobr Dabiré, Jeremy Bouyer, Abdoulaye Diabaté

**Affiliations:** 1 Institut de Recherche en Sciences de la Santé, Bobo-Dioulasso 01 BP 545, Burkina Faso; milabso@yahoo.fr (A.S.M.); kwamajacques@yahoo.fr (K.B.); bilgo02@yahoo.fr (E.B.); inoussatoe11@gmail.com (I.T.); dabireroch@gmail.com (R.K.D.); npiediab@gmail.com (A.D.); 2 Université Nazi Boni, Bobo-Dioulasso 01 BP 1091, Burkina Faso; 3 Centre Muraz, Institut National de Santé Publique, Bobo-Dioulasso 01 BP 390, Burkina Faso; 4 ASTRE, Cirad-Département BIOS, INRAE, Université de Montpellier, Plateforme Technologique CYROI, Sainte-31 Clotilde, 97490 La Réunion, France; jeremy.bouyer@cirad.fr

**Keywords:** mosquito control, sterile insect technique, males, deltamethrin

## Abstract

Background: Genetic control tools, such as the sterile insect technique (SIT) and genetically modified mosquitoes (GMMs), require releasing males comparable to their wild counterparts. Ensuring that released males do not exhibit higher insecticide resistance is critical. This study assessed the phenotypic characteristics and insecticide susceptibility of key dengue and malaria vector species. Methods: Phenotypic resistance to deltamethrin (0.05%) was tested in two-to-five-day-old male and female *Aedes aegypti* (Linnaeus, 1762) (Borabora and Bobo strains) and *Anopheles coluzzii* (Coetzee & Wilkerson, 2013) (Vallee du Kou strain) using WHO susceptibility guidelines. Wing measurements of live and dead mosquitoes were used to assess body size. Results: Mortality rates were similar between male and female *Ae. aegypti* (Bobo strain) and *An. coluzzii*, while *Ae. aegypti* Borabora was fully susceptible in both sexes. Females were consistently larger than males, with significantly larger live females than dead ones in the *Ae. aegypti* Bobo strain. Conclusion: This study highlights sex-specific differences in body size and insecticide susceptibility. Integrating these analyses into vector management programs is essential for the success and sustainability of SIT- and GMM-based interventions targeting malaria and dengue vectors. Implications for integrating genetic control strategies are discussed.

## Simple Summary

This study explored if laboratory male mosquitoes are as resistant to insecticides as females and how this could impact efforts to control diseases like dengue and malaria. Innovative methods, such as releasing sterilized or genetically modified male mosquitoes, depend on releasing males that can compete effectively with wild mosquitoes. Ensuring these males are not more resistant to insecticides than their wild counterparts is critical. Using WHO guidelines, we tested the resistance to deltamethrin in male and female *Aedes aegypti* mosquitoes from Bobo-Dioulasso and Borabora and *Anopheles coluzzii* mosquitoes from Vallée du Kou. We also measured their wing size to see if their body size affected their survival after insecticide exposure. The results showed that male and female *An. coluzzii* and *Ae. aegypti* from Bobo had similar levels of resistance. Females were generally larger than males, and in the Bobo strain, surviving females were larger than those that died. These findings highlight the importance of considering size and sex differences to ensure the success and sustainability of techniques like releasing sterilized or genetically modified mosquitoes for controlling mosquito-borne diseases.

## 1. Introduction

Mosquitoes transmit several pathogens of various diseases, including malaria, dengue, and Zika. Malaria and dengue are transmitted by *Anopheles* and *Aedes* mosquitoes, respectively. Insecticides are a common strategy for controlling mosquito populations [1]. However, the development of mosquito resistance to insecticides threatens to undermine these efforts. Resistance is most widespread in pyrethroids, the most commonly used class of insecticides [2,3,4,5,6]. The WHO expresses the urgent need for alternative methods of mosquito control that may be added to existing tools [7]. Alternative vector control strategies, including the sterile insect technique and other related techniques like genetic modification, have been tested [8,9] or are under development [10]. The sterile insect technique is based on the overflooding of a target population with released sterile males, inducing sterility in the wild female population. It has been shown to be effective against several insect pest species of agricultural and veterinary importance [11], and is under development for *Anopheles* and *Aedes* mosquitoes [9,12]. In the last decade, there have been several pilot release/mark–release–recapture studies using sterile, *Wolbachia*-infected mosquitoes or their combined-approach programs worldwide, including in Brazil [9,13], Malaysia [14], Sri Lanka [15], Singapore [16], and China [17,18], to name a few. In July 2019, the Target Malaria consortium released genetically modified sterile male *An. coluzzii* in Burkina Faso [19]. Implementing the sterile insect technique and related male-based release techniques begins with identifying a target vector, gaining knowledge of their behavior, and assessing the potential insecticide resistance in the colonized strain [9]. Several studies have looked at all aspects of mosquito biology and control, but males have been remarkably understudied [20], likely because they are not directly involved in disease transmission or targeted by insecticide spraying. More recently, given the renewed interest in genetic control techniques, aspects focused on male mosquito biology, including male mating behavior in the field [21,22] and competitiveness [12,23], were initiated.

Space spraying is recommended to control dengue outbreaks. Therefore, regular monitoring of susceptibility in the target population to insecticides used in public health is important for the development of new strategies to control dengue fever [24] and other related arbovirus-transmitting vectors. However, knowing that vectors develop multiple mechanisms of resistance to most classes of insecticides, it is essential to ensure that the released males are equally susceptible to insecticides as the wild population, to avoid the introduction of more resistant mosquitoes, while not killed at a higher rate than wild males by the insecticides which are applied in the release area. Little is known about the relative resistance level in males. One study found that male *Aedes albopictus* (Skuse, 1894) show higher mortality when exposed to insecticides in the WHO bioassay compared to females [25]. The authors showed that the susceptibility difference was due to several factors, including genetic susceptibility and body size [25]. A recent study highlighted that non-irradiated *Ae. aegypti* show changes in glutathione S-transferase (GST) activity in both male and female mosquitoes. Therefore, ionizing radiation increases GST activity, leading to a stress response, which may improve sterile male fitness when SIT and insecticides are combined [26]. Here, we aim to assess the phenotypic characteristics and insecticide susceptibility profiles of a pyrethroid in *Ae. aegypti* and *An. coluzzii* for future SIT or GM mosquito implementation in Burkina Faso.

## 2. Materials and Methods

### 2.1. Biological Material

Mosquito strains were maintained in the insectary of the Institut de Recherche en Sciences de la Santé (IRSS) in 2020, Bobo-Dioulasso, at a temperature of 27 ± 2 °C, a relative humidity of 75 ± 10%, and a light/dark cycle of L12:D12 h. Larvae were reared in dechlorinated tap water and fed TetraMin^®^ baby fish, while adults were fed a 10% (*w*/*v*) glucose solution ad libitum.

Two pyrethroid-resistant laboratory strains, including *Ae. aegypti* from Bobo-Dioulasso (Bobo strain) and *An. coluzzii* from Vallee du Kou (VK), Burkina Faso [27], were used for the experiments. A pyrethroid-susceptible *Ae. aegypti* strain (Borabora) originating from French Polynesia was also tested. Strains were maintained for about 3, 6, and 18 generations, for *Ae. aegypti* Bobo, Borabora, and *An. coluzzii* VK, respectively, before the experiments. The Borabora strain was provided by the French Institut de Recherche pour le Development (IRD), Montpellier, France.

### 2.2. Susceptibility Bioassay

Phenotypic resistance was assessed using two-to-five-day-old adult male and female mosquitoes of *Ae. aegypti* (Borabora and Bobo) and *An. coluzzii* VK, according to the standard WHO susceptibility test method [28]. During each test, two and four WHO test tubes of 25 ± 4 mosquitoes each were used as controls and treatments, respectively. The mosquitoes were initially acclimated for 1 h in acrylic recovery tubes (WHO kit). Both male and female mosquitoes originated from the same cohort. There were two test units per treatment within a biological replicate, which were technical replicates of each other. Deltamethrin-treated papers (0.05%) were purchased from Malaysia (University Sains Malaysia) as described in previous studies [3,4,5]. Mosquitoes were exposed to papers for 1 h and then kept with 10% glucose solution post-exposure in the same test conditions for 24 h to record mortality.

### 2.3. Mosquito Wing Length

Following the bioassays, live and dead mosquitoes of both *Ae. aegypti* Bobo and *An. coluzzii* VK strains were kept for wing length assessments. The live mosquitoes were anesthetized in a freezer (−20 °C) for five to ten minutes before dissection. Their right wings were dissected under a binocular magnifying glass using forceps. Each wing was photographed using LEICA EZ version 3.4.0 software (Leica Microsystems Holdings GmbH (LMS), Suisse, Deerfield, IL, USA). A 5 mm scale was also photographed for size reference. The size of the wings was measured from the annular notch to the end of the radius vein (excluding fringe scales) [29] with Image J version 1.5.3 software (Wayne Rasband, National Institutes of Health, Bethesda, MA, USA) using the “Measure” function.

### 2.4. Statistical Analyses

Statistical analysis was conducted using R version 4.2.3. To identify the difference in the mortality rates between male and female mosquito species treated with 0.05% deltamethrin, a binomial test for proportions at a 5% significance level was used. The non-parametric Kruskal–Wallis test was used to compare the distribution of mosquito wing size per sex, and the Wilcoxon test was used to analyze the differences in mosquito wing lengths between live and dead groups (males and females) under deltamethrin treatment.

## 3. Results

### 3.1. Susceptibility Bioassays

**Figure 1:**
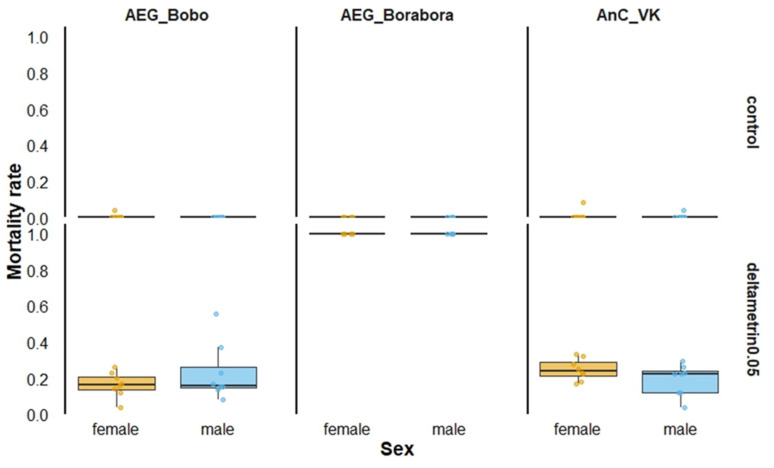
Twenty-four-hour mortality rates according to mosquito strain sex when exposed to 0.05% deltamethrin in World Health Organization tubes. AEG_Bobo = *Aedes aegypti* Bobo strain; AEG_Borabora = *Aedes aegypti* Borabora strain; AnC_VK = *Anopheles coluzzii* laboratory-resistant strain from Vallee du Kou. Controls were mosquitoes that were not exposed to deltamethrin-impregnated papers.

The pyrethroid-susceptible *Ae. aegypti* Borabora showed full susceptibility, resulting in 100% mortality when males and females were exposed to 0.05% deltamethrin (Figure 1).

Conversely, pyrethroid-resistant laboratory strains, including *Ae. aegypti* Bobo and *An. coluzzii* VK, exhibited between 16 and 25% overall mortality for both males and females. Males and females died similarly when the mortality was recorded 24 h after exposure in *Ae. aegypti* Bobo (Proportion test, χ^2^ = 2.6631, df = 1, *p* = 0.10) and in *An. coluzzii* VK (Proportion test, χ^2^ = 2.3721, df = 1, *p* = 0.12).

### 3.2. Mosquito Wing Length

**Figure 2:**
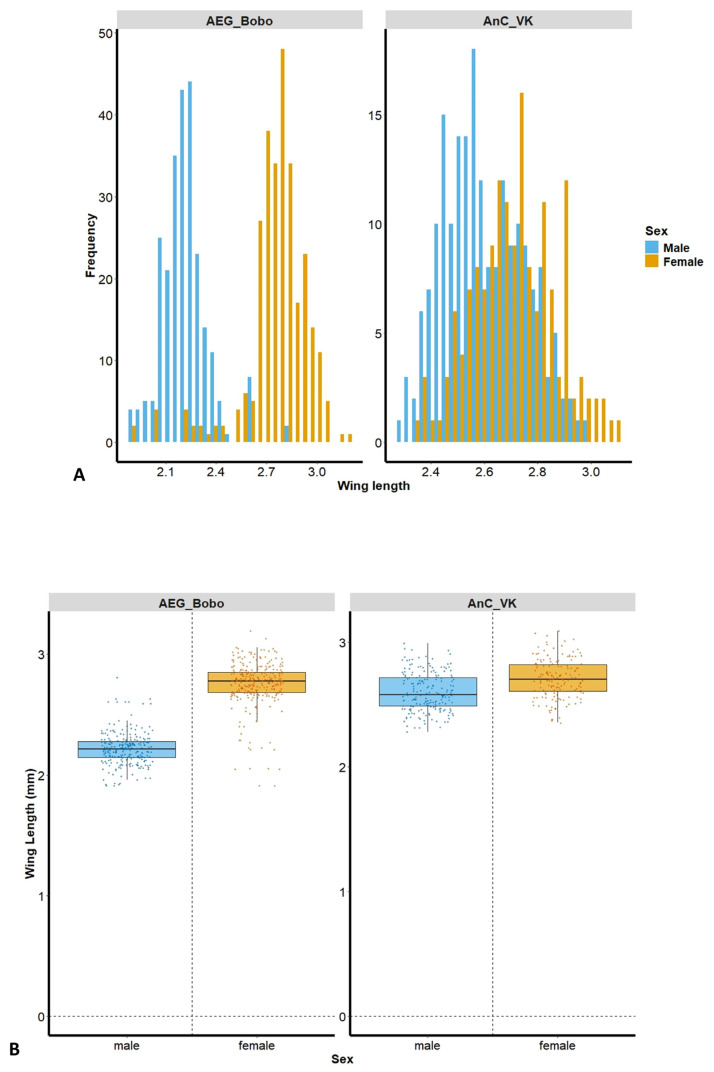
(**A**) Wing length distribution frequency according to mosquito strain and sex; (**B**) wing length of laboratory deltamethrin-resistant strains, *Aedes aegypti* Bobo (left panel) and *Anopheles coluzzii* laboratory-resistant strain from Vallee du Kou (VK) (right panel). AEG_Bobo = *Aedes aegypti* Bobo; AnC_VK = *Anopheles coluzzii* laboratory-resistant strain from Vallee du Kou (VK). Each dot represents a mosquito; the bars are the median mosquito wing length.

The size distribution frequency shows a greater separation between males and females in *Ae. aegypti* Bobo, and more overlapping lengths in *An. coluzzii* VK (Figure 2A). A total of male (*n* = 250) and female (*n* = 287) *Ae. aegypti* Bobo, as well as male (*n* = 197) and female (*n* = 158) *An. coluzzii* VK mosquitoes, were assessed. Female wing length was significantly greater than that of the males in *Ae. aegypti* Bobo (female: 2.75 ± 0.19 mm; male: 2.22 ± 0.14 mm, Kruskal–Wallis test: H = 342.184, *p* < 0.001) and in *An. coluzzii* VK (female: 2.71 ± 0.16 mm; male: 2.6 ± 0.15 mm, Kruskal–Wallis test: H = 37.443, *p* < 0.001) (Figure 2B).

**Figure 3:**
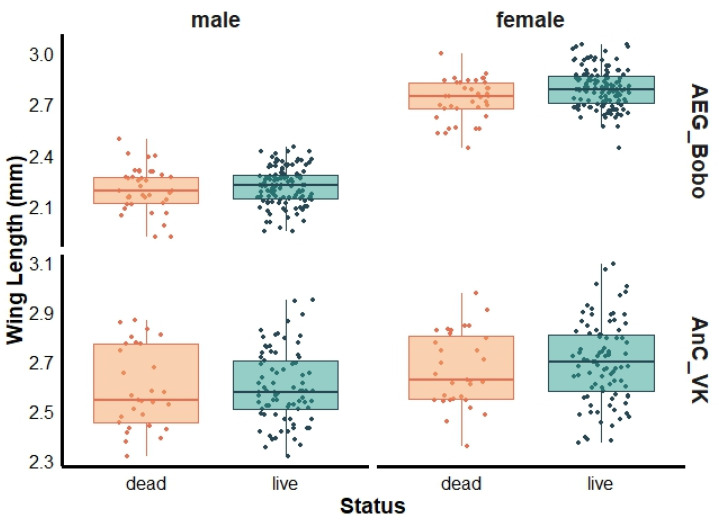
Wing length of dead and live females and males pyrethroid-resistant laboratory strains *Aedes aegypti* Bobo (AEG_Bobo) and *Anopheles coluzzii* Vallee du Kou (AnC_VK).

For female mosquitoes, a marginally greater wing length was observed in live than dead female *Ae. aegypti* Bobo (live: 2.77 ± 0.19 mm and dead: 2.74 ± 0.13 mm, Wilcoxon test, *p* = 0.04), with no significant size differences observed between live and dead males (live: 2.21 ± 0.11 mm and dead: 2.20 ± 0.12 mm, Wilcoxon test, *p* = 0.59) (Figure 3). The mean wing lengths of live and dead female *An. coluzzii* were 2.70 ± 0.16 mm and 2.67 ± 0.15 mm (Wilcoxon test, *p* = 0.37), respectively, and 2.60 ± 0.14 mm and 2.59 ± 0.16 mm (Wilcoxon test, *p* = 0.72) for live and dead males, respectively (Figure 3).

## 4. Discussion

Our study aimed to assess phenotypic characteristics and insecticide susceptibility profiles in dengue and malaria vector species, which are critical for effectively implementing genetic control tools like SIT and GMM releases. Ensuring released males have comparable resistance profiles to wild populations is desirable for the success and sustainability of these approaches when insecticides are used in release areas. We found similar patterns in *Ae. aegypti* and *An. coluzzii* laboratory strains. Males and females of both mosquito strains displayed similar mortality rates when exposed to 0.05% deltamethrin. There was a significant difference in length between live and dead female *Ae. aegypti* Bobo. Overall, female mosquitoes were larger than their male counterparts, as previously demonstrated [29,30]. Though it is not surprising because wing sexual dimorphism is prevalent in mosquito species, it highlights the importance of wing length in mosquito biology and its potential implications in vector control strategies.

The lack of significant differences in deltamethrin susceptibility between male and female *Ae. aegypti* Bobo and *An. coluzzii* VK strains aligns with earlier studies indicating that males typically exhibit resistance profiles similar to females in field populations [31]. Although the field-collected strains were maintained in the laboratory for about 3–18 generations before the bioassay, the uniform response to insecticide here suggests that body size may not influence deltamethrin susceptibility in the tested laboratory strains. Previous studies have indicated that physiological factors, such as detoxification enzyme activity and cuticle thickness, play a more significant role in pyrethroid resistance in *Anopheles* species [31,32]. The lack of size-based variability might also reflect a uniform distribution of these physiological traits between sexes in our laboratory-tested strains. Several other laboratory and field studies have shown contrasting results, with males being more susceptible than females. For instance, the ultra-low-volume (ULV) space sprays of malathion against *Ae. aegypti* in Venezuela, and the use of a pyrethroid formulation against *Ae. albopictus* (Skuse) in the United States, resulted in more detrimental effects on males [33,34]. Boubidi et al. [25] also reported that male *Ae. albopictus* were more susceptible in the laboratory due to their smaller size. Additionally, earlier research demonstrated that male mosquitoes tend to exhibit higher mortality rates than females [34]. The reported susceptibility of males may be attributed to multiple factors, including, first, physiological differences, as males typically exhibit lower detoxification enzyme activity compared to females, who rely on these enzymes to metabolize toxins encountered during blood digestion [35,36]. Second, body size and cuticle thickness contribute to these differences, with smaller male body size resulting in a higher surface-area-to-volume ratio, potentially increasing the effective dose of insecticide absorbed through the cuticle. Additionally, the larger body size and thicker cuticle of females may act as a barrier to insecticide penetration [37,38]. However, in our study, some behavioral factors may have affected the observed uniform susceptibility under deltamethrin. It is known that female mosquitoes, due to their host-seeking behavior, are more likely to encounter sub-lethal doses of insecticides. Therefore, more females may die due to repeated exposure. Males, which were less likely to contact treated surfaces, may have been less exposed to the insecticide and could have, therefore, survived better. Mortality rates can also differ among mosquitoes but may not explain the differences observed because a similar age group was used in our study [39]. Other studies have shown that resistant mosquitoes have fewer energy reserves than sensitive mosquitoes [40,41], implying that they use their energy reserves to activate processes that allow them to overcome toxins [42]. Field studies have reported high levels of resistance in wild *An. coluzzii* females to multiple insecticides, including pyrethroids and DDT [43] compared to laboratory-reared females, often bred in controlled environments without exposure to insecticides. A study comparing the susceptibility of wild and laboratory-reared *Aedes* and *Anopheles* larvae to ivermectin found that wild-derived larvae showed lower susceptibility compared to laboratory strains [44]. This suggests that lab-reared mosquitoes may lack the resistance mechanisms prevalent in wild populations, potentially compromising the effectiveness of SIT or GMM strategies if released males are disproportionately affected by insecticides used in the field.

Interestingly, the dead female size was smaller than that of the live female *Ae. aegypti* Bobo, suggesting that intra-group small and large individuals may have different tolerance to insecticides due to resistance mechanisms in the population. This increased susceptibility in smaller females is often attributed to their reduced energy reserves and lower levels of detoxification enzymes, which are crucial for metabolizing insecticides [35,36]. It is worth noting that major behavioral differences (activity patterns, flight, swarming, mating, resting, …) between aedine and anopheline mosquitoes could impact male survival differently, so implications for release programs may be species-specific.

The uniform susceptibility of male and female mosquito strains to deltamethrin may have implications for integrating genetic control strategies, such as SIT and GMM, with existing insecticide-based methods. This balance ensures that released males neither exacerbate resistance issues nor experience disproportionate mortality in insecticide-treated areas, enhancing program compatibility and effectiveness [45]. A combined approach, including insecticide and genetic tools, could enable simultaneous reductions in mosquito populations through complementary mechanisms [46]. Furthermore, it plays a critical role in resistance management by mitigating the risk of resistance allele spread or excessive male mortality, which could undermine intervention outcomes [31]. The findings also underscore the importance of routine resistance monitoring to ensure released males are phenotypically aligned with wild populations, supporting the long-term sustainability of genetic control programs [9,13]. The success of SIT or GMM programs largely depends on the ability of released males to survive and compete for mates in the wild. If released males are more susceptible to insecticides than their wild counterparts, their premature elimination can reduce the effectiveness of these interventions. Conversely, aligning the insecticide resistance profiles of released and wild-type mosquitoes can enhance the persistence and mating competitiveness of released males, thereby improving control outcomes. For instance, studies have shown that integrating biopesticides with SIT can significantly reduce the number of sterile males required to suppress mosquito populations [47,48]. Selvaraj et al. [49] simulated the reduced efficacy of current vector control measures in the presence of insecticide resistance and evaluated the likelihood of achieving local malaria elimination using gene drive mosquitoes released into a high-transmission setting alongside other vector control measures. They found that even a small number of insecticide-resistant vectors can lead to a rapid rise in malaria cases and that using gene drives in combination with traditional vector control is a more effective strategy for achieving malaria elimination compared to using either intervention alone. Another study developed a data-driven model to investigate the impacts of gene drive releases that cause vector population suppression on malaria prevalence and clinical cases in West Africa, showing that gene drive releases can substantially reduce malaria burden, especially when combined with other new interventions like RTS,S vaccines and pyrethroid-PBO bed nets [50].

Radiation-induced changes in enzymes, as reported by da Silva et al. [26], could further complicate SIT implementation. Their study highlights potential challenges in enzyme activation that may impact the fitness and resistance profiles of irradiated *Ae. aegypti* males, emphasizing the need for thorough pre-release evaluations. This also underscores the importance of optimizing irradiation protocols to ensure the competitive performance of released males in the wild. Assessing the susceptibility of irradiated/modified males to pyrethroids compared to non-irradiated/non-modified males may provide a comprehensive insight into the interaction between ionizing irradiation/modification and pyrethroid susceptibility.

## 5. Conclusions

This study highlights the uniform susceptibility of male and female *Ae. aegypti* Bobo and *An. coluzzii* VK laboratory strains to deltamethrin, suggesting that body size seems not to significantly influence insecticide resistance in these strains. These findings support the integration of genetic control tools, such as SIT or GMM, with insecticide-based interventions by ensuring compatibility and operational synergy. Additionally, the observed uniformity underscores the importance of routine resistance monitoring and optimization of irradiation protocols to maintain the fitness and alignment of released males with wild populations for the success and sustainability of genetic control programs in dengue and malaria vector management. Further studies may focus on genetically diverse populations and other insecticides with field and lab comparisons coupled with meta-genomic and metabolomic investigations to bring more insights into sex-specific insecticide resistance.

## Data Availability

The data supporting the findings of this study are available upon reasonable request.
